# 

**Published:** 1895-02

**Authors:** 


### CONTENTS FOR FEBRUARY, 1895.

ORIGINAL COMMUNICATIONS. PAGE.

Note on the Processes of Menstruation. By LAWSON TAIT, F. R. C. S. .... 385
A Case of Nephrectomy on Account of Nephrolithiasis and Pyonephrosis. By HERMAN MYNTER, M. D .... 388
Report of a Recent Case of Abdominal Pregnancy, Complicated with Pyosalpinx,—Laparatomy, Recovery. By H. T. WILLIAMS, M. D .... 392
Two Peculiar Cases of Hernia. By CLAYTON M. DANIELS, M. D .... 395

CLINICAL LECTURE.

Sarcoma of Tonsil—Cancer of Breast—Clinic at Buffalo General Hospital. By ROSWELL PARK, A. M., M. D .... 397

TRANSLATION.

The Treatment of Diphtheria with Antitoxin. By F. C. MOEHLAU, M. D .... 400

REPORTS OF SOCIETIES.

The Eighth Periodical International Ophthalmological Congress .... 402
Medical Society of the County of Erie .... 412

NEW INSTRUMENTS.

A Neurologist's Percussion Hammer .... 416

ABSTRACT.

Rules for the Prevention of Tuberculosis .... 418

EDITORIAL.

Antitoxin in Buffalo .... 420
Topics of the Month .... 423

---

Personal .... 427
Obituary .... 429
Medical College Notes .... 432
Book Reviews .... 433
Literary Notes .... 445
Miscellany .... 448

